# Seasonal variation of intra-ocular pressure in glaucoma with and without dry eye

**DOI:** 10.1038/s41598-020-70606-w

**Published:** 2020-08-18

**Authors:** Manami Kuze, Masahiko Ayaki, Kenya Yuki, Motoko Kawashima, Miki Uchino, Kazuo Tsubota, Kazuno Negishi

**Affiliations:** 1 Division of Ophthalmology, Matsusaka Central General Hospital, Matsusaka, Japan; 2 Department of Ophthalmology, Mie University School of Medicine, Tsu, Japan; 3 Department of Ophthalmology, Keio University School of Medicine, Tokyo, Japan; 4 Otake Clinic Moon View Eye Center, Yamato, Japan

**Keywords:** Corneal diseases, Glaucoma

## Abstract

The management of intra-ocular pressure (IOP) is important for glaucoma treatment. IOP is recognized for showing seasonal fluctuation. Glaucoma patients can be at high risk of dry eye disease (DED). We thus evaluated seasonal variation of IOP with and without DED in glaucoma patients. This study enrolled 4,708 patients, with mean age of 55.2 years, who visited our clinics in Japan from Mar 2015 to Feb 2017. We compared the seasonal variation in IOP (mean ± SD) across spring (March–May), summer (June–August), fall (September–November), and winter (December–February). IOP was highest in winter and lowest in summer, at 14.2/13.7 for non-glaucoma without DED group (n = 2,853, *P* = 0.001), 14.5/13.6 for non-glaucoma with DED group (n = 1,500,* P* = 0.000), 14.0/13.0 for glaucoma without DED group (n = 240,* P* = 0.051), and 15.4/12.4 for glaucoma with DED group (n = 115,* P* = 0.015). Seasonal variation was largest across the seasons in the glaucoma with DED group. IOP was also inversely correlated with corneal staining score (*P* = 0.000). In conclusion, the seasonal variation was significant in most of study groups and IOP could tend to be low in summer.

## Introduction

Glaucoma is the second leading cause of blindness worldwide^1^. Elevated intra-ocular pressure (IOP) is the only adaptable risk factor for glaucoma and the progression of visual field loss is strongly related to IOP. The management of glaucoma aims to reduce IOP^2,3^, which could be influenced by various factors including blood pressure, body posture, and diurnal and seasonal variations^4–6^. Mean IOPs are reportedly higher in winter and lower in summer in both normal^7–9^ and glaucoma^10,11^ subjects, and the magnitude of these fluctuations are larger in patients with glaucoma than in normal subjects. Clinically, it is important to control IOP since a large diurnal fluctuation in IOP is a known risk factor for the progression of glaucoma^12^. Seasonal and diurnal fluctuations could thus mislead a clinical decision regarding medication and surgery. Although this seasonal pattern was described two decades ago, the phenomenon is not completely characterised^8^.

Due to the chronic nature of glaucoma, many affected patients experience long-term exposure to commercial pharmaceutical components and preservatives that are known to cause corneal and conjunctival toxicity^13–15^. Previous studies have shown that up to 40% of glaucoma patients use more than one topical medication^16^, and benzalkonium chloride (BAK), the most common preservative in anti-glaucoma solutions, has been strongly implicated in inducing and/or exacerbating ocular toxicity such as corneal epithelial cell dysfunction and inflammatory and toxic side effects on the conjunctiva. Consequently, glaucoma-affected patients can be at high risk of also developing the commonly seen dry eye disease (DED)^17–19^.

DED also exhibits seasonal fluctuations in vulnerability against environmental conditions. DED signs and symptoms tend to vary throughout the year, and particularly with seasonal changes across summer to winter^8^. These findings corroborate the influential factors including climate, sunlight, temperature, humidity and light. The pathophysiology of DED is complicated since it is multifactorial, and there is dissociation between signs and symptoms in DED^20^. For instance, immunological, neurological, and psychological comorbidities could enhance the effect of seasonality on DED^8^.

We thus sought to understand the contribution of DED to seasonal variations in IOP, and herein we evaluated seasonal variation of IOP with and without DED in glaucoma patients.

## Results

**Figure 1:**
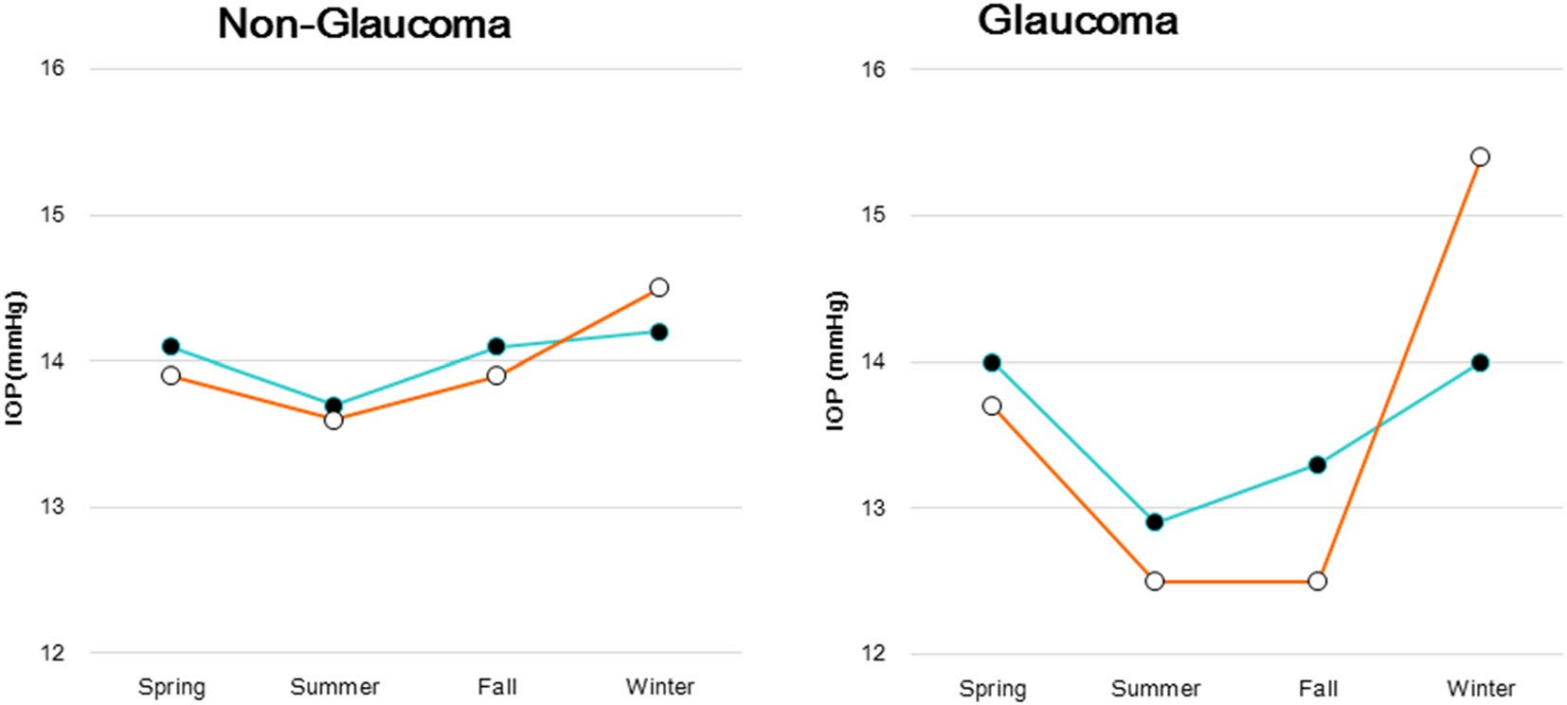
Seasonal change in mean IOP for patients with and without DED. The non-glaucoma group showed small seasonal amplitudes in both the DED (open symbol) and non-DED (closed symbol) groups (left panel). Glaucoma patients showed apparently large seasonal amplitudes in both groups, but especially in the DED group (right panel). The amplitude of seasonal variation was significantly larger in glaucoma patients with DED than in those without DE (*P* = 0.002, two-factor mixed design with repeated measures on one factor) and the non-glaucoma group with DED (*P* = 0.020). IOP; intra-ocular pressure, DED; dry eye disease.

Table 1 details the 4,708 consecutive patients enrolled in this study, with 1,692 males and a mean age of 55.2 years. The patient ophthalmological results are also listed in Table 1. Prostaglandin analogues (PG) are a first-line medication that was prescribed for 80.5% of non-DED cases and 73.3% for DED cases in this study. The averaged numbers of glaucoma medications being administered in this study cohort were 1.4 ± 0.6 for those with DED and 1.4 ± 0.6 for non-DED group. There was no difference in the severity of glaucoma between non-DED and DED groups. IOP was highest in winter and lowest in summer in all groups (Fig. 1). The IOP (mmHg, mean ± SD) levels in winter/summer and the resulting seasonal variation (winter-summer) were 14.2 ± 3.1/13.7 ± 3.0 and 0.5 for the non-glaucoma non-DED group (*P* = 0.001) compared to 14.5 ± 3.2/13.6 ± 3.1 and 0.9 for the non-glaucoma with DED group (*P* = 0.000), and 14.0 ± 3.3/13.0 ± 3.1 and 1.0 for the glaucoma non-DED group (*P* = 0.051) compared to 15.4 ± 6.2/12.4 ± 2.7 and 3.0 for the glaucoma with DED group (*P* = 0.015).

**Table 1:** Patient demographics and ophthalmological parameters.

	Non-glaucoma		Glaucoma		*P* value*
	Non-DED (n = 2,853)	DED (n = 1,500)	Non-DED (n = 240)	DED (n = 115)	
#(spring/summer/fall/winter)	608/819/838/588	377/402/426/295	56/82/62/60	23/30/28/34	
Age (year)	54.9 ± 18.6	53.3 ± 17.6	65.5 ± 13.7	63.2 ± 14.8	n.s
% male	42.3	19.6	56.3	37.1	< 0.001*
Refractive errors (diopter)	− 2.35 ± 3.14	− 2.49 ± 3.20	− 2.63 ± 3.52	− 3.12 ± 3.94	n.s
Mean IOP (mmHg)	14.0 ± 3.00	13.9 ± 3.1	13.5 ± 3.3	13.6 ± 4.4	n.s
**Glaucoma parameters**					
MD (dB)	–	–	− 5.11 ± 5.21	− 5.04 ± 6.06	n.s
Cupping (%)	–	–	72.0 ± 17.9	73.3 ± 16.8	n.s
GCC (μm)	–	–	85.7 ± 19.5	87.3 ± 21.4	n.s
Frequency of instillation	–	–	1.7 ± 1.2	1.9 ± 1.3	n.s
#of medication	–	–	1.4 ± 0.6	1.4 ± 0.6	n.s
**Glaucoma medication (%)**					
PG	–	–	80.5	73.3	n.s
Beta blocker	–	–	8.4	21.6	< 0.001
Fix (PG/beta blocker)	–	–	10.3	8.6	n.s
Fix (beta blocker/CAI)	–	–	12.3	14.8	n.s
CAI	–	–	9.6	11.2	n.s
Others	–	–	13.8	11.3	n.s
**DED parameters**					
Corneal staining score	0.21 ± 0.49	0.54 ± 0.74	0.37 ± 0.63	0.73 ± 0.82	< 0.001
BUT (s)	5.40 ± 1.49	2.72 ± 1.02	5.07 ± 1.65	2.73 ± 1.07	< 0.001

DED, dry eye disease; IOP, intra-ocular pressure; MD, mean deviation; GCC, thickness of ganglion cell complex; PG, prostaglandin analogue solution; Fix, fixed combination; CAI, carbonic anhydrase inhibitors BUT, tear break-up time; SPK, superficial punctate keratitis.

*P < 0.05, Non-DED versus DED with glaucoma, unpaired t test, or chi squared test as appropriate.

**Figure 2:**
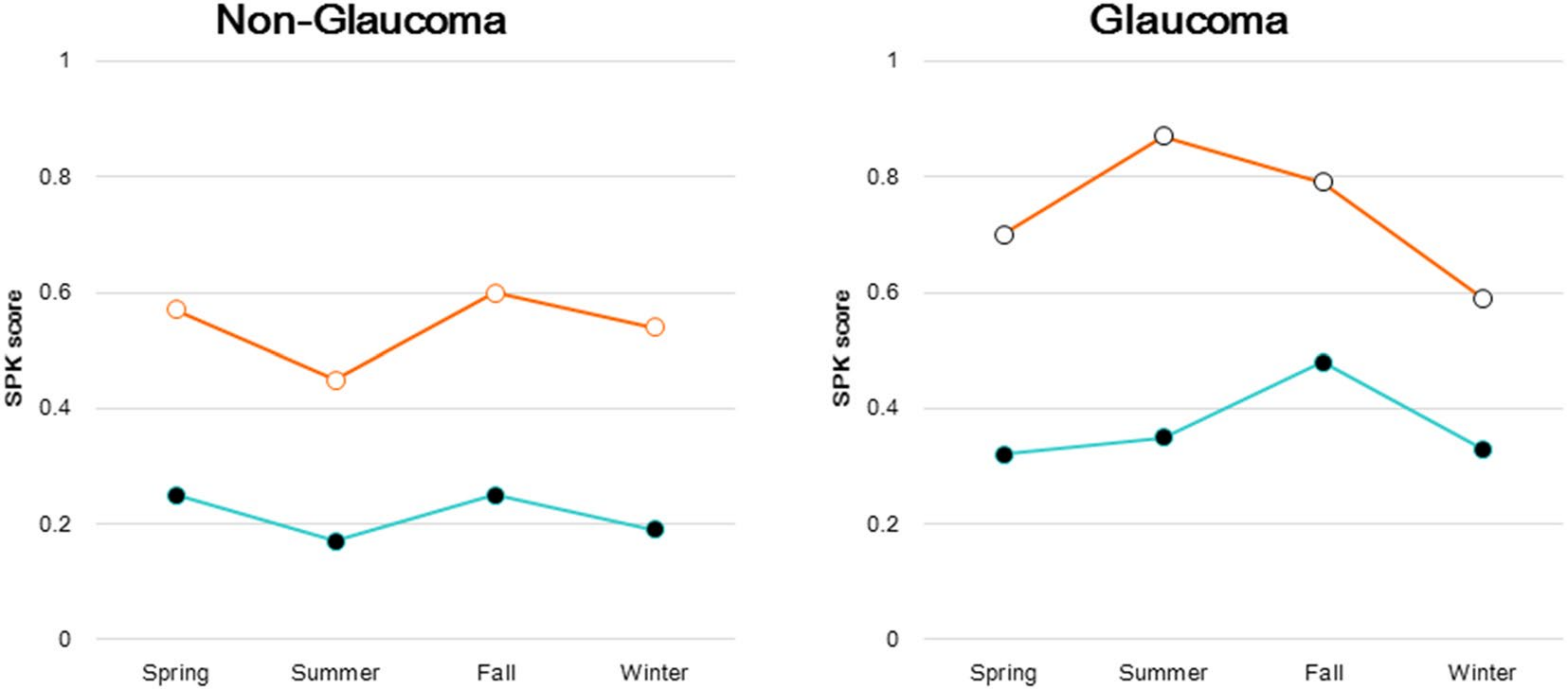
Seasonal change in SPK (superficial punctate keratitis) score for patients with and without DED. The non-glaucoma group showed small seasonal amplitudes in both the DED (open symbol) and non-DED (closed symbol) groups, with peaks in the fall and the lowest levels in summer (left panel). Glaucoma patients showed apparently large seasonal amplitudes in both groups, with the peak in summer for those with DED and in fall for those without DED (right panel). DED; dry eye disease.

**Figure 3:**
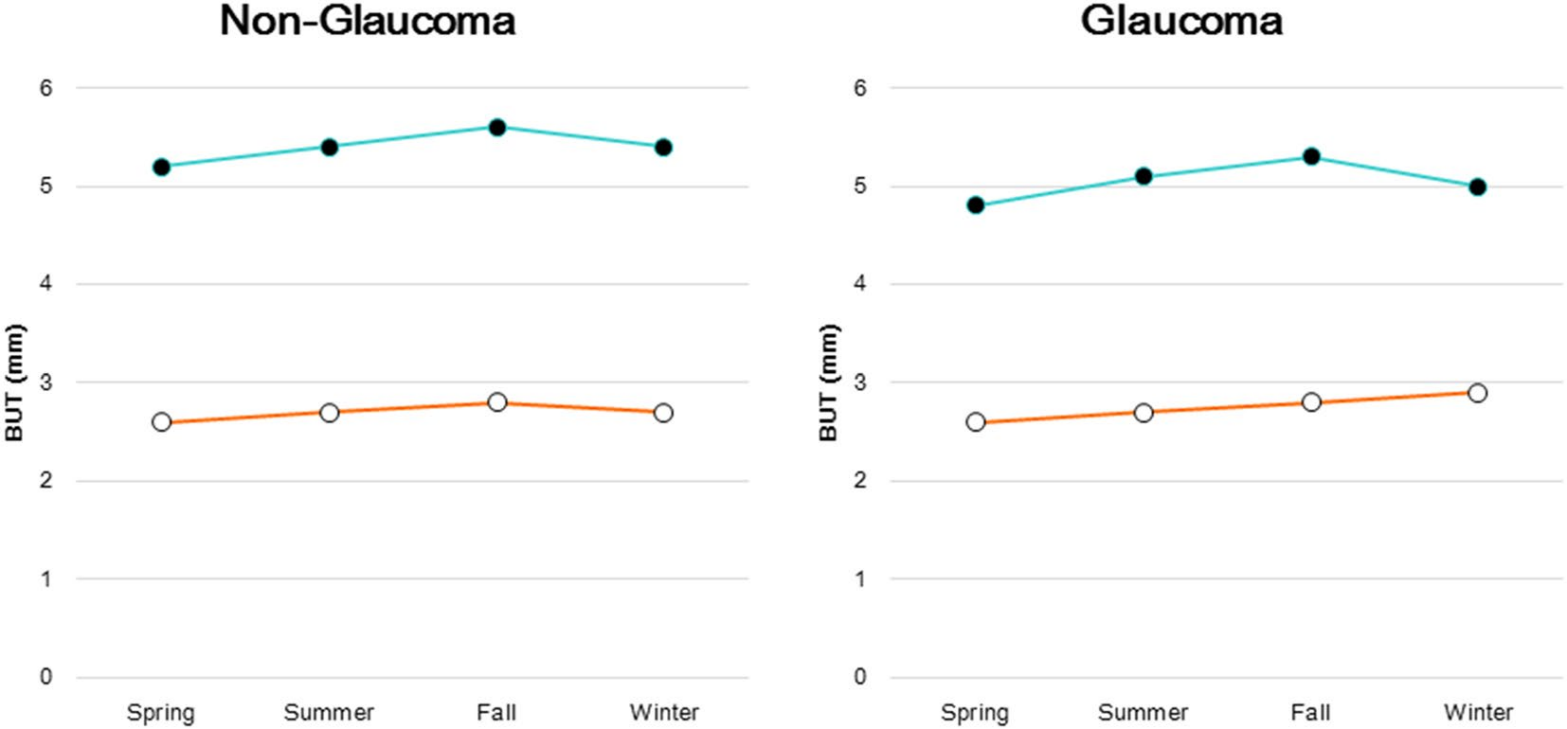
Seasonal change in BUT for patients with and without DED. There was no obvious seasonal amplitude observed in the non-glaucoma (left panel) or glaucoma group (right panel), with DED (open symbol) and without DED (closed symbol). BUT; tear break up time, DED; dry eye disease.

We conducted a two-way ANOVA for seasonal variation of IOP and found no significant difference between the non-glaucoma and non-DED group and the other three groups, and between the glaucoma and non-DED group and the other three groups (Table 2). The results of a one-way ANOVA indicated a significant seasonal variation in three groups. Regarding seasonal change of corneal staining score, the non-glaucoma group showed small seasonal amplitude changes with and without DED in both glaucoma groups, with the highest and lowest scores in fall and summer, respectively (Fig. 2). The glaucoma group showed apparently large seasonal amplitudes in both DED groups, with the peak of dry eye in summer and no dry eye in fall. Seasonal changes in BUT exhibited no obvious seasonal amplitude in any of the four groups (Fig. 3).

**Table 2:** The results of analysis of variance (ANOVA).

IOP (mmHg)	Seasons				Two-way ANOVA versus no glaucoma, non-DED	Two-way ANOVA versus Glaucoma, non-DED	One way ANOVA
	Spring	Summer	Fall	Winter			
No glaucoma, non-DED	14.1 ± 2.9	13.7 ± 3.0	14.1 ± 2.9	14.2 ± 3.1		Season: F = 1.555, *P* = 0.362 Glaucoma: F = 9.741, P = 0.052	F = 11.972 *P* < 0.001
No glaucoma, DED	13.9 ± 3.0	13.6 ± 3.1	13.9 ± 3.1	14.5 ± 3.1	Season: F = 0.703, *P* = 0.611 DED: F = 2.684, *P* = 0.200	Season: F = 0.720, *P* = 0.603 Glaucoma × DED: F = 4.246, *P* = 0.131	F = 4.470 *P* = 0.004
Glaucoma, non-DED	14.0 ± 3.7	13.0 ± 3.1	13.2 ± 3.2	13.0 ± 3.3	Season: F = 1.759, *P* = 0.327 Glaucoma: F = 0.069, *P* = 0.809		F = 1.781 *P* = 0.151
Glaucoma, DED	13.7 ± 4.2	12.4 ± 2.7	14.1 ± 5.1	15.4 ± 6.2	Season: F = 1.930, *P* = 0.301 Glaucoma × DED: F = 0.058, *P* = 0.825	Season: F = 0.885, *P* = 0.538 DED: F = 0.774, *P* = 0.443	F = 3.465 *P* = 0.019

IOP, intra-ocular pressure; DED, dry eye disease.

The regression analysis of 2,311 patients examined in both summer and winter identified seasonality (*P* < 0.0001) as the factor most strongly correlated with IOP, compared with presence of DED and glaucoma, corneal staining score, frequency of glaucoma eye drop instillation, and number of medications (Table 3). It is notable that severe SPK (high corneal staining score) tended to occur with low IOP (*P* = 0.050). Multiple regression analysis revealed that age and seasonality most affected IOP in summer and winter (Table 4).

**Table 3:** Regression analysis of IOP and various parameters.

Dependent valuables	IOP	SPK	BUT
Age (year)	− 0.271 (< 0.0001*)	− 0.052 (0.011*)	0.013 (0.513)
Sex^A^	− 0.004 (0.846)	− 0.171 (< 0.0001*)	0.255 (< 0.0001*)
Seasonality^B^	0.096 (< 0.0001*)	0.034 (0.096)	0.023 (0.244)
Glaucoma	0.006 (0.787)	0.115 (< 0.0001*)	− 0.050 (0.014*)
Frequency of instillation	− 0.010 (0.616)	0.145 (< 0.0001*)	− 0.038 (0.064)
# of medication	0.010 (0.615)	0.149 (< 0.0001*)	− 0.053 (0.010*)
MD	0.054 (0.244)	–	–
Cupping	0.014 (0.691)	–	–
GCC	0.056 (0.089)	–	–
DED	0.001 (0.949)	0.226 (< 0.0001*)	–
SPK	− 0.041 (0.050)	–	− 0.223 (< 0.0001*)
BUT	0.020 (0.352)	− 0.231 (< 0.0001*)	–

Data show β values, with *P*–values in parentheses. *P < 0.05, ^A^Male = 1, female = 0, ^B^Summer = 0, Winter = 1, all are adjusted for age and gender.

IOP, intra-ocular pressure; GCC, ganglion cell complex; MD, mean deviation; DED, dry eye disease; BUT, tear break-up time; SPK, superficial punctate keratitis.

**Table 4:** Multiple regression analysis with IOP.

Age (year)	− 0.262 (< 0.0001*)
Sex^A^	− 0.008 (0.712)
Seasonality^B^	0.096 (< 0.0001*)
DED	0.0001 (0.996)
Glaucoma	0.003 (0.870)

Data show β values, with *P*-values in parentheses. *P < 0.05, ^A^Male = 1, female = 0, ^B^Summer = 0, Winter = 1, all are adjusted for age and gender.

IOP, intra-ocular pressure; DED, dry eye disease.

## Discussion

Our results are compatible with previous studies of a seasonal fluctuation in IOP^21,22^ wherein IOP was higher during the winter than the summer by 0.5 mmHg for non-glaucoma patients without DED and by 1.0 mmHg for glaucoma patients without DED. Glaucoma medications might not contribute to this fluctuation since they tend to instead reduce the magnitude of diurnal fluctuations^23,24^. The advantage of the present study lies in the data being obtained from individual cases in a sufficiently large population covering all age groups without overlapping for 2 years. The status of DED varies across seasons if repeatedly examined so that this study design seems to work well to adequately classify the participants into groups with and without DED. We accordingly examined each patient once to immediately correlate IOP with DED, and future studies should be carried out in a standard method with repeated examinations on the same patients to adequately analyze seasonal variation.

We speculate a potential mechanism of seasonal fluctuations in IOP driven by three factors. The first is an autonomous nervous system (ANS) as conventionally hypothesized^25,26^ accounting for high IOP in winter. The second is SPK induced by BAK^27^, and the third is corneal thinning. The ANS regulates many systemic functions including body temperature, heart rate, and ocular function in the form of pupil diameter, accommodation, and IOP^28^.

Primary open-angle glaucoma is characterized by a large diurnal IOP change compared to controls and is typically induced by ANS dysregulation^25,26^. Koga and Tanihara suggested that ANS and/or cathecholamic tone could influence the IOP via vascular tone in blood pressure^29^. Furthermore, the high prevalence of comorbidity with abnormal ANS in DED patients might amplify the seasonal fluctuation in IOP^30^.

The severity of SPK was marginally correlated with IOP being lowest in summer, suggesting that increased drug permeability might enhance the pressure reduction effect. Tight junctions in the superficial cells of corneal epithelium are damaged by BAK contained in glaucoma medications^31^, which causes the junctions to degrade. As a consequence, the normal barrier function of the cornea suffers^32^. After exposure to BAK, the conjunctival epithelium can also directly undergo apoptosis. The corneal permeability generally corresponds to the concentration of BAK^33^ and also depends on the property of other ingredients^34^. However, the direct correlation between corneal permeability and actual pressure reduction effect has not been determined and some experimental results are contradictory with respect to SPK facilitating the intra-ocular penetration of anti-glaucoma solutions^35^. Thirdly, apparently low IOP in summer might be caused by corneal thinning^36^. The central corneal thickness decreases in DED^37^ and contributes to low IOP in summer, since measured IOP might be lower than the actual value. Ophthalmologists should be also aware that perimetric results could thus be overestimated in summer^38^ because the sensitivities in summer were 0.2 dB below those of winter/spring.

The limitations of our study include the regional and systemic background of participants, because both DED and IOP are closely associated with climate, temperature, humidity, ambient light, and various systemic parameters that should have been fully evaluated and adjusted for analysis. IOP shows normal diurnal variation and should therefore also be measured at a fixed schedule. We used a cross-sectional design, whereby each eye was tested only once, limiting the statistical power. A more powerful study design would involve measuring IOP in the same cohorts of glaucoma and non-glaucoma patients multiple times across the four seasons, so that the actual seasonal variation can be quantified and compared between groups. Our present study partially addressed such a limitation by including a large sample size. Diagnosis of glaucoma mostly depended on the clinical decision of each participating ophthalmologist rather than following strict diagnostic criteria, although classification of DED was clearly based on appropriate criteria and we were able to achieve reasonable results. Lastly, participants with ocular hypertension without medication would have better served as controls.

In conclusion, the seasonal variation was significant in most of study groups and IOP could tend to be low in summer. Possible explanations include ANS dysfunction, corneal thinning, and corneal change caused by DED and anti-glaucoma medication. We recommend noting to patients that IOP might be lowest in summer and highest in winter especially in patients with DED. We examined each patient once to correlate IOP with a timely onset of DED; however, standard practice also demands repeated examinations using the same patients to properly analyze seasonal variation.

## Methods

### Study participants and Institutional Review Board approval

We conducted a cross-sectional, case-control study in Komoro Kosei General Hospital (Nagano, Japan), Shinseikai Toyama Hospital (Toyama, Japan), Tsukuba Central Hospital (Ibaraki, Japan), Jiyugaoka Ekimae Eye Clinic (Tokyo, Japan), Todoroki Eye Clinic (Tokyo, Japan), and Takahashi-Hisashi Eye Clinic (Akita, Japan). We consecutively recruited participants visiting these eye clinics from March 2015 to February 2017. Exclusion criteria were visual impairment (< 20/25 in either eye), any ocular surgery within the last three months, or age under 20 years.

The latitude in the Tokyo area on mainland Japan is 35.68° North and day length varies by 4–6 h over the year according to averages for 1981–2010 reported by the Japan Meteorological Agency. Japan is characterized by four distinct seasons with marked variations in temperature, humidity, and daylight time. Generally, Japan is hot and humid in summer and cold and dry in winter. The Japan Meteorological Agency reported average temperatures and humidity from 5.2 °C and 52% in winter to 25.0 °C and 77% in summer in the Tokyo area in the years 1981–2010.

This study was approved by the Institutional Review Board and Ethics Committee of Keio University School of Medicine, Komoro Kosei General Hospital, Tsukuba Central Hospital, and Jiyugaoka Ekimae Eye Clinic and was conducted in accordance with the Declaration of Helsinki. Informed consent was obtained from all participants.

### Ophthalmological examinations

All cases were diagnosed as bilateral open-angle glaucoma or normal-tension glaucoma under slit lamp biomicroscopy. Glaucoma were diagnosed through routine examinations and a visual field test (Humphrey Visual Field Analyzer 30-2 standard program; Carl Zeiss, Jena, Germany), measuring the thickness of ganglion cell complexes using optical coherent tomography (OCT; RC3000^R^) (Nidek, Gamagori, Japan and Cirrus® HD-OCT (Carl Zeiss, Jena, Germany)). Diagnostic criteria for glaucoma comprised glaucomatous visual field loss in the Glaucoma Hemifield Test, an ophthalmoscopic neurofiber layer defect, a cup/disc ratio > 0.6, or elevated IOP (> 21 mmHg) requiring topical medication for more than six months. Patients were excluded if they had retinal pathology, retinal surgery, photocoagulation affecting the visual field or coexisting cataract with significant lens opacity disturbing the optical axis that accounted for subjective visual disturbance or decreased visual function. Topical glaucoma medication included Xalatan® (Pfizer, Tokyo, Japan), Tapros® (Santen Pharmaceutical Co. Ltd., Osaka, Japan), Travatanz® (Alcon Laboratories, Tokyo, Japan), Lumigan® (Senjyu Pharmaceutical Co. Ltd., Osaka, Japan). Xalacom® (Pfizer), Tapcom® (Santen Pharmaceutical Co. Ltd), Duotrav® (Alcon Laboratories), Aifagan (Senjyu Pharmaceutical Co. Ltd.), Detantol® (Santen Pharmaceutical Co. Ltd.), Nipradinol® (Kowa), Mikelan® LA (Otsuka), and 0.5% Timoptol® XE (MSD).

DED was diagnosed according to the criteria of the Asia Dry Eye Society^39^: presence of DED-related symptom(s) and a tear break-up time (BUT) shorter or equal to 5 s. We also conducted the Schirmer test^40^, and measured maximum blinking interval (≤ 9 s) and superficial punctate keratoepitheliopathy (staining score ≥ 3).

### Ophthalmological examinations

BUT was measured using a wetted fluorescein filter paper (Ayumi Pharmaceutical Co Ltd, Tokyo, Japan) applied at the lower lid margin. To ensure adequate mixing of the fluorescein dye with the tear film, subjects were instructed to blink three times. The interval between the third blink and appearance of the first dark spot in the cornea was measured three consecutive times using a stopwatch. For this study, we considered the means of the three measurements as the BUT. We scored the ocular surface in three sections (nasal conjunctiva, cornea, and temporal conjunctiva) on a scale of 0–3 for severity. Overall epithelial damage was graded on a scale of 0–9^41^.

### Statistical analysis

Participants were assigned to one of four groups based on the presence of glaucoma and/or DED. The season on the date of each patient’s visit was classified as follows: spring from March to May, summer from June to August, fall from September to November, and winter from December to February. The effect of season on IOP was assessed by one-way analysis of variance (ANOVA), and the interaction was assessed using a two-way ANOVA. Regression analysis was performed to explore which parameters most affect seasonal difference in IOP. Data are presented as the mean ± standard deviation (SD) or as percentages where appropriate. All analyses were performed using StatFlex^R^ (Atech, Osaka, Japan), with *P* < 0.05 considered significant.
